# Use of Peptides for the Management of Alzheimer’s Disease: Diagnosis and Inhibition

**DOI:** 10.3389/fnagi.2018.00021

**Published:** 2018-02-07

**Authors:** Mohammad H. Baig, Khurshid Ahmad, Gulam Rabbani, Inho Choi

**Affiliations:** Department of Medical Biotechnology, Yeungnam University, Gyeongsan, South Korea

**Keywords:** Alzheimer’s disease, peptides, amyloid beta, BACE-1, GAPDH, inhibitors, diagnosis

## Abstract

Alzheimer’s disease (AD) is a form of dementia and the most common progressive neurodegenerative disease (ND). The targeting of amyloid-beta (Aβ) aggregation is one of the most widely used strategies to manage AD, and efforts are being made globally to develop peptide-based compounds for the early diagnosis and treatment of AD. Here, we briefly discuss the use of peptide-based compounds for the early diagnosis and treatment of AD and the use of peptide-based inhibitors targeting various Aβ aggregation checkpoints. In addition, we briefly discuss recent applications of peptide-based inhibitors against various AD targets including amyloid beta, β-site amyloid precursor protein cleaving enzyme 1 (BACE1), Glyceraldehyde-3-phosphate dehydrogenase (GAPDH), tyrosine phosphatase (TP) and potassium channel KV1.3.

## Introduction

Alzheimer’s disease (AD) is the most common fatal neurodegenerative disease (ND), and is characterized by the structural and functional loss of neurons. During the last few decades, AD and its associated risk factors have become major healthcare concerns in most developed countries. Furthermore, it has been reported AD is the fifth-leading cause of death among those aged more than 65 years, and that its incidence exceeds five million cases per year in the United States (Alzheimer’s Association, 2017). The World Health Organization (WHO) estimated that the prevalence of AD worldwide will quadruple to reach approximately 114 million by 2050 (Alzheimer’s Association, 2017).

Pathological hallmarks of AD include the progressive accretion of plaque outside neurons (extracellular amyloid plaque) and of neurofibrillary tangles inside neurons (hyperphosphorylated tau protein accumulations; VanItallie, 2015). These pathological changes gradually result in neuronal loss and eventual neuron death. Although the etiology and pathogenesis of AD remain imprecise, the amyloid cascade theory is widely accepted and supported by several studies (Drachman, 2014; Herrup, 2015). This hypothesis was further reinforced by the identification of a protective amyloid precursor protein (APP) mutation near the protein’s beta-cleavage site, which protects against the development of late onset dementia (Jonsson et al., 2012).

Amyloid-beta (Aβ) is a 39–43 amino acid residue peptide and a key component of extracellular amyloid plaque, and its expression is considered a key event in AD progression (Li et al., 2017). Aβ is the peptide product of the sequential proteolytic cleavages (by β- and γ-secretases) of APP (a type-I transmembrane protein). These proteolytic cleavages result in the generations of two types of Aβ isoforms (Aβ40 and Aβ42), and though Aβ40 is more abundant than Aβ42 in human fluids, Aβ42 aggregates faster and is considered to be the more lethal in terms of neuron survival. It has been reported oligomers of diffusible Aβ, such as, protofibrils, prefibrillar aggregates and Aβ-derived diffusible ligands (ADDLs), are the main toxic players during the development and progression of AD (Haass and Selkoe, 2007; Shankar et al., 2008; Funke and Willbold, 2012). Numerous amyloid reduction therapy (ART) clinical trials have failed to provide expected clinical improvements in AD patients, and these failures raise genuine concerns regarding the validity of the amyloid cascade hypothesis and the merits of further research on ART (Extance, 2010; Grundman et al., 2013; Cheng et al., 2017).

Today, due to the extensive efforts of researchers and pharmaceutical companies, peptide-based drugs have emerged as a major class of therapeutics, and as a result, the last decade has witnessed extraordinary scientific and industrial interest in their therapeutic uses (Vlieghe et al., 2010). Such studies are very much on-going and currently, a number of natural and synthetic therapeutic peptides are undergoing clinical trials (Mandal et al., 2014). Generally, these drugs have several advantages over small molecule therapeutics, particularly in terms of their efficacies and fewer side effects (Craik et al., 2013).

Many other fatal NDs, such as, Parkinson’s disease (PD), amyotrophic lateral sclerosis (ALS), prion diseases and Huntington’s diseases (HD) share the AD characteristic of misfolded protein aggregation. Peptides have proven to be vital tools for ND research, and can be used to study the properties of misfolded proteins and/or peptides. In this review article, we discuss some of the available peptide-based therapeutics used to treat AD, the use of peptide inhibitors to target different aspects of AD, and the use of peptides for the diagnosis and early detection of AD.

## The Use of Peptides as Diagnostic Probes

Currently, the number of *in vivo* diagnostic techniques available for detection of AD is limited, but early detection of the disease is crucial for effective treatment, because preemptive treatment might control or eliminate early stage disease. Investigations on AD patients have shown amyloid plaque appears several years before cognitive symptoms (Silverman et al., 1997; Thal et al., 2002; Funke and Willbold, 2012). Accordingly, early stage detection and the quantification of amyloid in brain are viewed as important from the prognostic point of view and for evaluating the effects of therapies, and several molecular imaging techniques, including, magnetic resonance imaging (MRI), positron emission tomography (PET) and single-photon emission computed tomography (SPECT) provide means of doing so. These molecular imaging techniques have been reported to be useful for detecting biomarkers of AD (mainly Aβ) and for monitoring the expression of Aβ (Frisoni et al., 2017), and thus, have attracted considerable attention in the AD research field. Furthermore, amyloid ligands have been used as contrast agents to estimate amyloid plaque load, and been shown to stain amyloid plaque specifically in the brain tissues of AD patients, which make these peptides suitable probes for *in vivo* imaging (Kang et al., 2003).

D-enantiomeric peptides (also known as derivatives of ACI-80) are another set of peptides that have been reported to bind Aβ1–42. In one study, ACI-80 also was found to bind Aβ1–42 with high affinity (in the submicromolar range; Funke et al., 2012; Gulyás et al., 2012), and is currently being used as a molecular probe to monitor Aβ1–42 plaque load in the living brain. Findings show when ACI-80 is injected into the brain, it specifically binds to Aβ1–42 and stains dense amyloid deposits in brain but not diffuse plaque (van Groen et al., 2009), which makes it a suitable molecular probe for *in vivo* imaging in AD. In addition, recent studies have described a series of D-enantiomeric peptides that also specifically bind to aggregated Aβ1–42 (Funke et al., 2012), but have greater stabilities and Aβ binding properties than ACI-80. To confirm binding by these peptide derivatives, *ex vivo* immunochemistry was performed using transgenic mouse models of AD, and the ACI-80 derivatives ACI-87-Kφ, ACI-88-Kφ and ACI-89-Kφ were found to bind to aggregated Aβ1–42 with greater affinity than parent ACI-80. These findings suggest these compounds might be useful probes for specific types of Aβ aggregation and plaque *in vivo*.

Larbanoix et al. (2010) used a phage display technique to search for peptide ligands with carrying ability to vectorize an AP-targeting contrast agent, and identified 12 peptide ligands from among 22 sequenced phage clones with high affinity against Aβ42. Two peptides were selected (C-FRHMTEQ-C and C-IPLPFYN-C) as both were found to be present in several copies and to have K_d_-values in the picomolar range. For example, C-IPLPFYN-C was found to have a K_d_ value of 2.2 × 10^−10^ M, and C-FRHMTEQ-C a K_d_ value 5.45 × 10^−10^ M against mouse Aβ42. Both peptides were also tested on human Aβ42, and C-FRHMTEQ-C demonstrated similar affinity against human Aβ42, whereas C-IPLPFYN-C demonstrated greater affinity against human than mouse Aβ42. In addition, a preliminary *in vivo* MRI study on a transgenic mouse model of AD, showed both C-FRHMTEQ-C and C-IPLPFYN-C acted as excellent contrast agents (Larbanoix et al., 2010).

## Use of Peptides as Inhibitors Against Alzheimer’S Disease

### Peptide Inhibitors of Amyloid β

**Figure 1:**
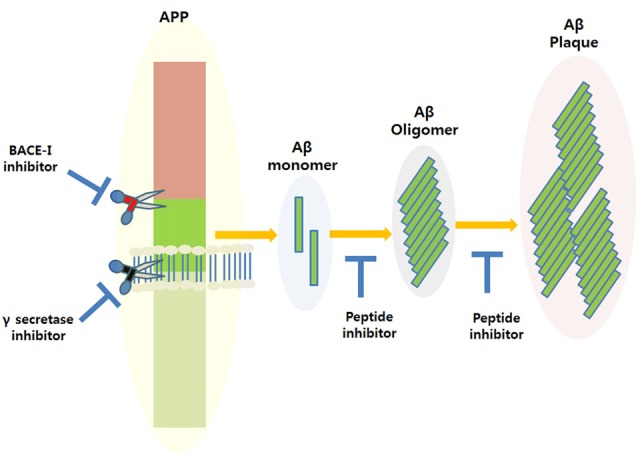
Amyloidogenic amyloid precursor protein (APP) processing and the possible applications of peptide inhibitors to prevent amyloid-beta (Aβ) plaque formation at different disease stages.

Amyloid plaque accumulation and Aβ fibrillation are clinical hallmarks of AD (Hajipour et al., 2017), and thus, the inhibition of amyloid aggregation has been the subject of much research over the last two decades, and the use of peptide-based inhibitors represents a major part of these efforts (Goyal et al., 2017; Folch et al., 2018; Figure 1). A large number of these studies have focused on the design of peptide fragments capable of binding to Aβ regions critical for aggregation (Ladner et al., 2004). The peptides designed function by binding to Aβ to either prevent fibril formation or Aβ elongation to prevent the formation of monomers/oligomers. A long list of peptides that specifically target Aβ have been designed (Doig, 2007; Eskici and Gur, 2013), but here, we discuss some of the more recently reported peptides inhibitors of Aβ. Table 1 shows some of the important peptides described in the literature as inhibitors of AD. Peptide inhibitors that share sequence similar with hydrophobic segments of Aβ are capable of altering Aβ aggregation and reducing the cytotoxicity of Aβ (Wasmer et al., 2008). Austen et al. (2008) described two peptide inhibitors of Aβ, that is, RGKLVFFGR (OR1) and RGKLVFFGR-NH2 (OR2), which were produced by modifying the KLVFF amino acid sequence of Aβ by incorporating RG−/−GR residues at its N- and C-terminals. These inhibitors were both reported to effectively inhibit Aβ fibril formation.

**Table 1:** List of peptide-based inhibitors reported to be effective against Alzheimer’s disease (AD).

Peptides	Description	Reference
RGKLVFFGR (OR1) RGKLVFFGR-NH2 (OR2)	Based on the Aβ (16–20) sequence	Austen et al. (2008)
Fc-KLVFF	Based on the Aβ (16–20) sequence and conjugated with ferrocenoyl (Fc)	Wei et al. (2011)
KLVF-ΔA-I-ΔA KF-ΔA-ΔA-ΔA-F	Based on the Aβ (16–20) sequence and ΔAla	Wei et al. (2011)
RYYAAFFARR	Based on Aβ (11–23)	Liu et al. (2014)
KLVFFA, KKLVFFA, KFVFFA, KIVFFA and KVVFFA	Based on the Aβ (16–20) sequence and replacement of L- with D-amino acids	Chalifour et al. (2003)
PGKLVYA, KKLVFFARRRRA and KKLVFFA	d-peptides of Aβ (16–20)	Jagota and Rajadas (2013)
AuNPs@POMD-pep	Aβ peptide conjugates with gold nanoparticles (AuNPs)	Gao et al. (2015)
IGLMVG-NH2	Based on Aβ (32–37)	Bansal et al. (2016)
HKQLPFFEED	A β-sheet blocker peptide based on the stereochemical structure and characteristic of aggregation of Aβ (1–42)	Lin et al. (2014)
Nonhemolytic 11-residue peptide (NAVRWSLMRPF)	Modified analog of NIVNVSLVK from the E-protein sequence of SARS coronavirus	Ghosh et al. (2017)
AP90	A 23 residue long hairpin peptide (with alternating D- and L-amino acids) reported to inhibit Aβ aggregation.	Kellock et al. (2016)

In another study, Wei et al. (2011), reported some new peptide inhibitors based on modifications of the hydrophobic KLVFF amino acid sequence of Aβ, and synthesized a conjugate of the pentapeptide KLVFF and ferrocenoyl (Fc) to improve the lipophilicity and proteolytic stability of peptide inhibitors. In another study, Rangachari et al. (2009) designed two α, β-dehydroalanine (ΔAla) containing peptides based on KLVFF, and found two novel ΔAla-containing peptides (KLVF-ΔA-I-ΔA and KF-ΔA-ΔA-ΔA-F) disrupted Aβ aggregation, though by quite different mechanisms.

Another group of researchers designed and synthesized a decapeptide inhibitor of Aβ1–40 aggregation. This inhibitor (RYYAAFFARR) was designated RR, and unlike other inhibitors was designed to target the extended region of Aβ (Aβ11–23), which consists of a GAG-binding site, a hydrophobic core, and a salt bridge region. RR was found to have high binding affinity for Aβ1–40 (*K*_d_ = 1.10 μM), and its binding affinity was markedly greater than that of the known β-sheet breaker peptide iAβ5 (*K*_d_ = 156 μM; Liu et al., 2014).

Chalifour et al. (2003) reported results obtained by replacing L-amino acids with D-amino acids with the aims of increase peptide stability and therapeutic potential. The effect of chiral reversal (D-enantiomers) was assessed for five peptides (KKLVFFA, KLVFFA, KIVFFA, KFVFFA and KVVFFA), and it was found they inhibited Aβ aggregation better than L-peptides. In particular, the D-enantiomer of KKLVFFA was found to inhibit the neurotoxic effect of Aβ significantly more than its L counterpart.

Jagota and Rajadas (2013) synthesized three short D-peptides, that is, KKLVFFARRRRA, PGKLVYA and KKLVFFA, based on residues of the central hydrophobic core of Aβ (residues 16–20), and examined their effects on Aβ aggregation. Observations suggested these D-peptides effectively inhibited Aβ fibrillogenesis, and two of the three (KKLVFFA and PGKLVYA) were found to improve survival in transgenic *C. elegans*.

### β-Site APP Cleaving Enzyme 1 (BACE1)

Beta-site APP cleaving enzyme 1 (BACE1) is a human aspartyl protease, which is believed to play a prime role in the generation of beta amyloid peptides (Aβ) in AD. BACE1 has characteristic bilobal structure and is a membrane-bound aspartyl protease with an open active site, which is less hydrophobic than those of other aspartic proteases and allows up to 11 substrate residues to be accommodated (Hong et al., 2000; Turner et al., 2005). BACE1 levels are elevated in the brain tissues of AD patients and its overexpression in cerebrospinal fluid offers a possible biomarker of early stage disease. When BACE1 is overexpressed it competes with γ-secretase to initiate the cleavage of APP at its β-position. Furthermore, BACE1 inhibition halts Aβ formation at the first step of APP amyloidogenic processing. BACE1 is composed of two signature peptides, that is, DTGS at position 93–96 and DSGT at position 289–292, which come together to form an active site and have the ability to inhibit APP (Yan et al., 2016). Peptides derived from the sequence of BACE1 have also been reported to inhibit APP processing. BACE1 possess a catalytic domain containing a pair of aspartic acid residues at its active site, and Aβ production is blocked by BACE1 inhibition, which reduces Aβ production by depleting C99, acting as a substrate of γ-secretase. The observation that Aβ production was diminished in BACE1 deficient mice supports the view that BACE1 inhibitors reduce Aβ levels (Vassar, 2002).

### Glyceraldehyde-3-Phosphate Dehydrogenase (GAPDH)

Glyceraldehyde-3-phosphate dehydrogenase (GAPDH) is a glycolytic enzyme of considerable interest in ND research, especially in AD. Recently, it was revealed that GAPDH interacts with APP, which is known to be involved in AD (Sunaga et al., 1995; Bertram et al., 2007; Butterfield et al., 2010). GAPDH can undergo diverse oxidative modifications that control its structure, function, and activity, and in AD, when exposed to oxidative stress Aβ forms amyloid-like aggregates, which reduce neuron and synapse numbers. Furthermore, insoluble aggregates of GAPDH accelerate Aβ amyloidogenesis and neuronal cell death *in vitro* and *in vivo* (Itakura et al., 2015), and GAPDH aggregation caused by cysteine oxidation and intermolecular disulfide bonding reduces its catalytic activity (Nakajima et al., 2007, 2009). Glycyl-L-histidyl-L-lysine-Copper (GHK-Cu) is naturally occurring peptide in human plasma with a stunning array of actions that appear to counter aging-associated diseases and conditions. In plasma the concentration of GHK-Cu is about 200 ng/ml (10^−7^ M^−1^) at age 20, but decreases to 80 ng/ml at age 60 (Pickart et al., 2015), and interestingly, the enzyme primarily involved in GAPDH gene silencing also belongs to the histone deacetylase (HDACs) protein family. Selective HDAC inhibitors have been shown to possess neuroprotective properties in animal models of brain disease, and have been suggested as potential therapeutics for AD (Fischer et al., 2010). Gly-Pro-Glu (GPE) is present in plasma and brain tissues, and it has been shown to have neuroprotective effects in animal models of NDs, such as, HD, PD and AD (Alexi et al., 1999). Basic structural studies suggest that GPE can interact with single or several Glu receptor types and bind to N-methyl-D-aspartate (NMDA) receptor (Sara et al., 1989). Furthermore, the C-terminal (Glu) GPE is required for NMDA receptor binding and this binding induces the potassium-evoked release of dopamine from nigrostriatal dopaminergic terminals and acetylcholine through an unknown mechanism via NMDA receptor (Sara et al., 1989; Alonso De Diego et al., 2005).

### Tyrosine Phosphatase (TP)

STriatal-Enriched tyrosine phosphatase (STEP) is expressed in neurons of the striatum, neocortex, hippocampus and related structures (Pelkey et al., 2002), and targets signaling pathways in the postsynaptic terminals of excitatory glutamatergic synapses (Lombroso et al., 1991; Boulanger et al., 1995). STEP regulates various synaptic actions including glutamate receptor trafficking, which plays critical functions in learning and memory (Karasawa and Lombroso, 2014). Interestingly, it was recently suggested STEP is overactive in AD and schizophrenia (Carty et al., 2012). Endogenous STEP levels also influence the susceptibility of neurons to excitotoxicity and affect the regulation of synaptic proteins by changing synaptic conductivities via the synchronized dephosphorylations of multiple substrates that regulate synaptic plasticity. STEP is an intracellular tyrosine phosphatase (TP) encoded by the *ptpn5* gene, and contains a signature consensus sequence [I/V]HCxAGXXR[S/T]G at its C-terminus that is required for its catalytic activity (Bult et al., 1996), and the active motif (I/V)HCXAGXGR(S/T), also called the P-loop, which houses catalytic Cys for nucleophilic attack is a hallmark of the PTP super-family. Furthermore, a neuroprotective, endogenous tripeptide GPE was reported to protect and rescue cells from Aβ-induced death, and modified analogs of GPE, including modifications at Pro and/or Glu residues, have been synthesized and evaluated (Guan and Gluckman, 2009). Binding of NNZ-2566 (glycyl-L-2-methylprolyl-L-glutamic acid) analogs with Aβ was found to have better neuroprotective effects in infant rats, as compared with GPE (Cacciatore et al., 2012).

### Peptide Based Inhibitors Against Potassium Channel KV1.3

Potassium channel KV1.3 was recently identified as a potential target in AD (Rangaraju et al., 2015; Lowinus et al., 2016). Rangaraju et al. (2015) conducted a study on AD and non-AD patients and found Kv1.3 overexpression in the frontal cortices of AD patients, thus suggesting potassium channel KV1.3 to considered a therapeutic target in AD. BmKTX-R11-T28-H33 (ADWX-1), OsK1-K16-D20 and HsTx1 [R14A] are examples of peptides subsequently designed to target Kv1.3 (Norton and Chandy, 2017).

## Conclusion and Future Perspectives

A large amount of research effort has resulted in advancements in the diagnosis and treatment of AD, the etiology of which is considered by most to be explained by the amyloid cascade hypothesis. Furthermore, it is generally considered the early detection of AD is likely to facilitate its treatment using advanced therapeutic approaches, and that the use of peptides for the diagnosis of AD offers an effective means of doing so. Nevertheless, these peptide-based approaches require further development to enable early AD to be efficiently diagnosed and properly treated.

## Author Contributions

IC and MHB conceived the idea. MHB, KA and GR drafted the review manuscript. IC, MHB, KA and GR critically reviewed the article. IC edited the language and corrected the errors within the manuscript.

## Conflict of Interest Statement

The authors declare that the research was conducted in the absence of any commercial or financial relationships that could be construed as a potential conflict of interest.

## Abbreviations

Aβ: Amyloid-beta
AD: Alzheimer’s disease
BACE1: β-site APP cleaving enzyme 1
GAPDH: Glyceraldehyde-3-phosphate dehydrogenase
ND: Neurodegenerative disease
TP: Tyrosine phosphatase.
